# No difference in the level of sports activity between single versus dual mobility total hip arthroplasty in adults: a clinical trial

**DOI:** 10.1186/s40001-025-02470-1

**Published:** 2025-03-28

**Authors:** Riccardo D’Ambrosi, Filippo Maria Anghilieri, Federico Valli, Giovanni Palminteri, Guido Bandettini, Baldo Arcuri, Ilaria Mariani, Laura Mangiavini, Nicola Ursino, Filippo Migliorini

**Affiliations:** 1 IRCCS Orthopedic Institute Galeazzi, Milan, Italy; 2 Dipartimento di Scienze Biomediche per la Salute, Università degli Studi di Milano, Milan, Italy; 3 Institute for Maternal and Child Health-IRCCS “Burlo Garofolo”, Trieste, Italy; 4 Department of Orthopaedic, Trauma, and Reconstructive Surgery, RWTH University Hospital, Pauwelsstraße 30, 52074 Aachen, Germany; 5 Department of Orthopaedic and Trauma Surgery, Academic Hospital of Bolzano (SABES-ASDAA), 39100 Bolzano, Italy; 6 Department of Life Sciences, Health, and Health Professions, Link Campus University, 00165 Rome, Italy; 7 Scuola Di Specializzazione in Ortopedia e Traumatologia, Università Degli Studi Di Milano, Milan, Italy

**Keywords:** Hip arthroplasty, Dual mobility, Return to sport, Physical activity

## Abstract

Current evidence on the quality of sports activity in patients younger than 65 following dual mobility (DM) total hip arthroplasty (THA) is lacking, and whether this coupling allows better performance than single mobility (SM) still needs to be fully clarified. This clinical trial compared sport-related patient-reported outcome measures (PROMs) of the traditional SM versus DM implants in active patients younger than 65. All THAs were performed using a minimally invasive posterolateral approach, polyethylene liner and ceramic head. All implants were cementless. The University of California, Los Angeles (UCLA) activity scores, the High-Activity Arthroplasty Score (HAAS), the visual analogue scale for pain (VAS), and the Harris Hip Score (HHS) were administered to each patient. Patient assessment was conducted on admission, at 12, and at a minimum of 24 months postoperatively. A total of 403 patients were included in the study: 372 SM and 31 DM. The mean age was 56.3 ± 7.2 years. The mean length of the follow-up was 51.3 ± 21.0 months. The present clinical trial found no difference in UCLA, HHS, HAAS, and VAS. Patients returned at a similar level of sports activity in both groups.

**Level of evidence** Level II, prospective group-controlled clinical trial.

## Introduction

Total hip arthroplasty (THA) is commonly performed in orthopaedics [1]. Patients undergoing THA have become younger and, more frequently, actively participate in recreational activities [2, 3]. The most significant increase in THAs is observed in patients younger than 65, especially those aged 45 to 54 [4]. Patients younger than 65 years are commonly involved in recreational activities, and a new challenging quest is to allow patients to return to practice increasingly demanding sports activities. Return to sports after total joint arthroplasties was thought to be related only to patient demographics and surgeon recommendations [5, 6]. Recent advantages in THA in tissue-sparing approaches and biomaterials have been introduced to reduce wear and creep to offer a faster and safer return to sports [7–9]. Restrictions in hip range of motion (ROM) and hip instability are two essential causes of sports retirement following THA [10]. To overcome the risk of these complications, dual mobility (DM) THA has been introduced. Unlike traditional single mobility (SM) implants, DM incorporates an additional using a mobile polyethylene liner. However, current evidence on the quality of sports activity in patients younger than 65 years following DM is lacking, and whether this implant allows better performance of SM still needs to be fully clarified. Therefore, this clinical trial compared SM versus DM in sport-related patient-reported outcome measures (PROMs) in active patients younger than 65.

## Methods

### Study protocol

The procedures involving humans in this study adhered to the ethical standards of the Declaration of Helsinki and its later amendments and were approved by the Ethics Committee of the San Raffaele University Hospital of Milan, Italy (CE 236/2017). The present study was conducted following the STROBE checklist [11]. All the participants signed written informed consent. The present study was conducted at the Department of Orthopaedics of the IRCCS Orthopaedic Institute Galeazzi, Milan, Italy, between 2013 and 2019.

### Surgical procedures and rehabilitation protocol

Patients were allocated based on their preferences for a specific surgeon. Patients who chose one surgeon (N.U.) underwent SB, while those who chose another (F.V.) received DB. All patients received a 1.5-g single shot of intravenous cefuroxime and 1-g of intravenous tranexamic acid during incision. A minimally invasive posterolateral approach in a lateral decubitus was used in all patients irrespectively of the implant used. A delta ceramic and high-density crosslinked polyethylene were used in the SD group. A Trilogy Cup (Zimmer-Biomet, Warsaw, Indiana, USA) and a Fitmore Hip Stem (Zimmer-Biomet, Warsaw, Indiana, USA) were used. In the DM group, the Jump Traser System (Permedica Orthopaedics, Merate, Lecco, Italy) with large polyethylene liner (Permedica Orthopaedics, Merate, Lecco, Italy), ceramic head (Permedica Orthopaedics, Merate, Lecco, Italy) and Synthesis femoral stem (Permedica Orthopaedics, Merate, Lecco, Italy) were used. A six-week anti-thrombotic prophylaxis with 4000 UI daily of enoxaparin was administered. No drainages were used. Indomethacin 100 mg twice daily was administered to prevent heterotopic ossification. Both surgeons followed the same physiotherapy regimen.

The postoperative rehabilitation regimen involved full weight-bearing ambulation under the observation of physiotherapy using crutches immediately on postoperative day one. The functional active and passive motion was allowed with an initial restriction on intra-rotation and adduction for a week. An abduction pillow was used for the first two weeks. The average time until the patients were discharged was 5.6 days. 85% (343 of 403) of patients were discharged to an inpatient physiotherapy clinic for two weeks, and 15% (60 of 403) underwent outpatient physiotherapy. The “European Hip Society” recommendations were followed for the return to sport [6]. Briefly, most physical activities were allowed for the patients 6 months after THA. The patient's experience performing a distinct sport activity did not influence the recommendations to return to former sports activities.

### Eligibility criteria

The inclusion criteria were: (1) symptomatic end-stage hip osteoarthritis stadium II to III, according to the Tönnis classification; (2) patients being able to understand the nature of the treatment. The exclusion criteria were: (1) chronic or acute inflammatory diseases; (2) neoplastic diseases; (3) pregnancy; (4) immunodeficiency; (5) severe peripheral neuropathy; (6) osteoporosis or other bone ailments which require stem and cup cementation; (7) other omitted criteria which may have influenced the results of the present investigation.

### Clinical evaluation

The clinical evaluation was performed by two assessors with long experience in sports medicine and arthroplasty surgery who were not involved in the clinical management of the patients. The following PROMs were administered: the University of California, Los Angeles (UCLA) activity scores, High-Activity Arthroplasty Score (HAAS), the visual analogue scale for pain (VAS), and the Harris Hip Score (HHS) [12–15]. Patient assessment was conducted on admission, at 12, and at a minimum of 24 months postoperatively. Data concerning the following complications were also collected: peri-prosthetic fractures and infections, dislocations and revision surgeries.

### Statistical analysis

An overall cohort of 381 patients was estimated to be adequate to detect a 0.5 mean difference in subsequent measurements of HAAS score, given a standard deviation of 3, a 0.05 type I error, and a 0.90 power, using a two-tailed paired t-test [16]. Additionally, 22 subjects were recruited to ensure statistical significance in case of loss at follow-up by adverse events. Sample characteristics are represented as absolute numbers, percentages, or means and standard deviations (SDs). Group comparisons were performed using IBM SPSS version 25. To compare the PROMs, the mean differences effect measure was adopted. The t-test was performed, with values of P < 0.05 considered statistically significant.

## Results

### Demographic data

A total of 403 patients were included in the study: 372 SM and 31 DM. The mean age was 56.3 ± 7.2 years, and 57% were male. The mean follow-up was 51.3 ± 21.0 months. Detailed results are reported in Table 1.

**Table 1:** Demographic data

Endpoint	Total (n = 403)
Women	175 (43.4%)
Mean age	56.3 ± 7.18
Age ≥ 60yrs	162 (40.2%)
Mean BMI	27.6 ± 4.16
Normal weight (BMI < 24.9)	116 (28.7%)
Overweight (BMI 24.9 to 29.9)	169 (41.9%)
Grade I obesity (BMI 30 to 34.9)	97 (24.1%)
Grade II obesity (BMI 35 to 39.9)	21 (5.2%)
Follow-up (months)	51.3 ± 21.0
Surgery length (minutes)	105.1 ± 16.1

*SD* standard deviation, *BMI* Body Mass Index, *DM* dual mobility, *SM* standard mobility

### Baseline comparability

No differences were found between the groups at baseline (Table 2).

**Table 2:** Group comparison at baseline

Endpoints	SM (n = 372)	DM (n = 31)	*P*
Women	41.9% (156 of 372)	61.3% (19 of 31)	0.06
Age	56.2 ± 7.3	57.0 ± 6.4	0.6
BMI	27.6 ± 4.2	27.3 ± 4.1	0.9
Surgery length (minutes)	104.9 ± 15.8	107.6 ± 20.1	0.8
UCLA	4.2 ± 1.3	4.2 ± 1.2	0.8
HHS	45.1 ± 8.6	45.2 ± 9.2	0.8
HAAS	5.0 ± 1.3	5.0 ± 1.1	0.9
VAS	8.3 ± 1.0	8.4 ± 1.0	0.8

*SD* standard deviation, *BMI* Body Mass Index, *DM* dual mobility, *SM* standard mobility, *UCLA* University of California, Los Angeles, *HAAS* High-Activity Arthroplasty Score, *VAS* visual analogue scale for pain, *HHS* Harris Hip Score

### Results synthesis

No difference was found in UCLA, HHS, HAAS, and VAS between SM and DM at 12 months (T_1_) and at a minimum of 24 months (T_2_) of follow-up (Table 3).

**Table 3:** Comparison of clinical outcomes groups

PROM	SM (n = 372)	DM (n = 31)	*P*
UCLA			
T_1_	6.5 ± 1.2	6.6 ± 0.8	0.7
T_2_	6.6 ± 1.1	6.6 ± 0.9	0.9
HHS			
T_1_	89.9 ± 6.5	90.2 ± 5.1	0.9
T_2_	90.1 ± 6.2	91.4 ± 4.2	0.3
HAAS			
T_1_	11.8 ± 1.8	11.9 ± 1.2	0.1
T_2_	12.3 ± 2.1	12.4 ± 1.2	0.1
VAS			
T_1_	1.6 ± 1.2	1.4 ± 1.0	0.6
T_2_	1.6 ± 1.1	1.35 ± 0.8	0.4

*SD* standard deviation, *UCLA* University of California, Los Angeles activity scores, *HAAS* High-Activity Arthroplasty Score, *VAS* visual analogue scale for pain, *HHS* Harris Hip Score, *DM* dual mobility, *SM* standard mobility

### Complications

Two complications occurred in the SM group: one dislocation (reduced in the emergency room) and one acute infection, which was managed with surgical debridement, antibiotics, and implant retention. No statistically significant difference was found in complications between the two groups (p > 0.05).

## Discussion

Patients younger than 65 who have undergone both SM or DM returned at a similar level of sports activity at approximately 51 months of follow-up.

Hip instability after THA has become a concern. Recent studies conducted on a large scale reported that dislocation accounts for up to 25% of the cause of failure [17–19]. Patient features can significantly increase the risk of postoperative dislocation, particularly in the presence of abductor deficiency, acute fracture, psychiatric problems, and neurological disease. Moreover, native anatomy or pathological anatomy deformed by the disease process may be a challenge for the surgeon, making it difficult to obtain stability of the implant with standard implants [20]. For all these matters, in recent years, dual mobility implants have gained much popularity among hip surgeons. From a biomechanical perspective, the outer diameter of the liner provides a jump distance that theoretically prevents implant dislocation once the operated tissues around the hip joint have healed [10, 21]. In patients with a relatively small size (acetabular diameter < 50 mm), surgeons are constrained to an implant with a limited head–neck ratio, a restricted range of motion and an increased risk of dislocation. Adding this bone morphology to obesity and a large inner thigh produces a ‘cocktail’ for dislocation [10, 22]. Dual-mobility designs involve two distinct articulation regions: the first between the femoral head and the polyethylene liner and the second at the interface between the convex surface of the polyethylene liner and the acetabular shell. The primary articulation is between the femoral head and the polyethylene liner and is involved in most activities with standard range-of-motion requirements [23]. The secondary articulation between the polyethylene liner and the acetabular shell is engaged. At the same time, activities that exceed the normal range of motion, especially when the neck of the femoral stem contacts the rim of the liner, should be avoided. These two articulations allow for a greater range of motion, a greater head-to-neck ratio, and a more physiologically effective head size, which enhances the jump distance and, hence, forms a resistance to dislocation. Computational models have shown an increased range of motion with dual mobility versus traditional implants. Additionally, a greater distance-to-impingement imparted by the dual articulations correlates with decreased impingement and subsequent dislocations [24]. Guyen et al. [25] experimentally evaluated the range of motion to impingement of dual-mobility implants with 22.2 mm and 28 mm femoral head sizes. The dual-mobility implants authors evidenced an increased range of motion compared with standard implants, reporting increased flexion of 30.5°, adduction of 15.4°, and external rotation of 22.4° [25]. A systematic review published in 2018 analysing 10,783 DM THA found that the incidence of aseptic loosening was 1.3% (142 hips), the rate of intraprosthetic dislocation was 1.1% (122 hips), and the incidence of extraarticular dislocation was 0.46% (41 hips). The overall survivorship of the acetabular and dual mobility components was 98.0%, with all-cause revision as the endpoint at a mean follow-up of 8.5 years (2 to 16.5) [26].

Returning to sports after THA is frequently limited by subjective recommendations made by surgeons to avoid failures and concerns possibly associated with physical activity. Generally, surgeons wish to give recommendations based on sound evidence, but the literature on this topic remains limited. The major sports-related concerns after THA are implant survival, instability, periprosthetic fracture, and implant wear [27]. However, surgical techniques have been modified and improved over the past decades, and muscle-sparing techniques have become increasingly popular. In addition, using larger heads and developments in biomaterials and coating methods, such as ultra-high molecular weight crosslinked polyethylene and high-performed ceramics, is believed to prolong implant survival [28, 29]. More than 30% of patients listed for THA practice sports routinely, and returning to their activities is a major concern. However, the risk of premature failure is a concern. The hip is put through 5.5 times the body weight during jogging, which results in a 43% increase in contact stresses within the prosthetic hip joint [30]. Elevated wear rates are often linked to sports that require increased activity levels, leading to osteolysis and reduced implant survival. Exposing THA to high torque forces is linked to elevated wear rates and periprosthetic fracture. Moreover, there is a risk of dislocation in sports that require a wide range of hip movement at the extremes of motion [31]. However, a previous study has shown that 61.4% of patients return to practice sports within one to three years following THA; while this group is self-selected, some evidence suggests that patients involved in high-activity/impact sports achieve higher outcome scores [32]. Despite technically successful surgery, the risk of failing patients’ expectations about returning to sports after arthroplasty is reflected in poor outcome scores. Hence, counselling THA patients adequately before surgery and addressing their expectations is critical [32]. Recently, two surveys were performed to better analyse the return to sports after THA [6, 33]. In 2016, British Hip Society members were explored through a 12 web-based survey [33]. There were 109 responses from a total of 260 people who were surveyed. Most respondents (33%) were interested in performing uncemented procedures, 29.1% were interested in hybrid procedures, 15.5% were interested in fully cemented procedures, and 11.7% wanted to execute a resurfacing hip arthroplasty for sporting patients [33]. The most preferred advance is the standard posterior (68.9%), while the most preferred bearing couples are ceramic-on-ceramic (39.8%) and ceramic-on-polyethylene (36.9%) [33]. Half of the respondents believed they would choose a femoral head smaller than 36 mm, whereas 22.3% of the respondents thought they would use a head of 36 mm or a larger head. At least one-third of the respondents believed they would allow patients to return to sports between six and twelve weeks following surgery [33]. In contrast, 43.7% of the respondents advised patients to wait for three months after the operation [33]. All respondents allowed the patients to return to low-impact activities; however, notable care must be practised when performing high-impact activities [33]. Most recently, European Hip Society (EHS) members were invited to complete an online questionnaire, including recommendations for 47 sports disciplines [6]. 150 (32.9%) EHS members participated in the survey [6]. The participants believed that five sports activities were enough after six weeks of THA [6]. Furthermore, participants agreed that ten activities can be performed after six to 12 weeks of surgery [6]. Likewise, 26 activities can be performed after three to six months of surgery [6]. After six months of surgery, 37 of the 47 activities may be executed. High-intensity activities, such as handball, soccer/football, basketball, full-contact sports and martial arts, were not allowed after the surgery [6].

The present study has several limitations. The THAs were conducted in a high-volume tertiary hospital and performed by two surgeons well beyond their learning curve. The length of the follow-up was approximately 50 months, which might jeopardise long-term outcome comparability. Additional studies with longer follow-ups are necessary. Moreover, patient activity levels may change beyond the follow-up, increasing data variability in longer follow-ups. Despite the overall sample size being adequate and powered, the two groups were unbalanced (372 SM and 31 DM); the DM group was relatively smaller than the SM, which might reduce the generalizability of the study. Moreover, the authors used two implants of two different enterprises. Despite both implants being certified and approved for THA, whether differences in the outcomes exist between implants is unclear. Zimmer Biomet is a leading global medical device company specialising in designing, manufacturing, and marketing orthopaedic products and related surgical solutions. Zimmer Biomet provides a wide range of products and services primarily focused on musculoskeletal health, which includes joint replacement implants, surgical instruments, and other orthopaedic and biologic solutions. Permedica Orthopaedics is an Italian company that develops, produces, and distributes high-quality orthopaedic implants and instruments. They provide solutions for joint replacement surgeries, such as hip, knee, and shoulder. Permedica Orthopaedics is well-known for its innovation in materials and design, aiming to enhance patient outcomes and improve the durability of its products.

## Conclusions

Patients younger than 65 who have undergone both SM or DM returned at a similar level of sports activity at approximately 51 months of follow-up.

## Data Availability

All data and materials are available on reasonable request to Dr. Riccardo D’Ambrosi (riccardo.dambrosi@hotmail.it).

## Abbreviations

THA: Total hip arthroplasty
SM: Single mobility
DM: Dual mobility
UCLA: University of California, Los Angeles
HAAS: High-Activity Arthroplasty Score
VAS: Visual analogue scale
HHS: Harris Hip Score
